# Book Review: The New Paradigm of Immunity to Tuberculosis

**DOI:** 10.3389/fimmu.2013.00386

**Published:** 2013-11-18

**Authors:** Shawn D. P. Ellis, Elliott R. Carthy

**Affiliations:** ^1^Department of Medicine, University College London, London, UK; ^2^School of Medicine, Imperial College London, London, UK

**Keywords:** *Mycobacterium*, vaccination strategies, regulatory T cells, tuberculosis, book review

The treatment of tuberculosis presents an enormous challenge to global health. Tuberculosis is a leading cause of death from infectious diseases worldwide, second only to the human immunodeficiency virus (HIV). Although several therapeutics and vaccines are available to treat and prevent tuberculosis infections, more virulent varieties of tuberculosis and increased drug resistance in the form of multi, extreme, and total drug resistant disease are major emergent threats. *Mycobacterium tuberculosis* is an obligate human pathogen and has co-evolved with humans for millennia, thus it has influenced the immune system of individuals who live in tuberculosis endemic regions. It is essential that therapeutic and research strategies targeted at tuberculosis should reflect this co-evolution. Elucidating the basic biology and immunology of tuberculosis is relevant to increasing and consolidating our fundamental understanding of the disease; thus, facilitating the development of therapeutics that will eradicate the disease and enable the immune system to achieve protection from tuberculosis.

The book, *The New Paradigm of Immunity to Tuberculosis* edited by Maziar Divangahi (1), seeks to address the need for a comprehensive understanding of the immunological processes underlying tuberculosis infection by combining immunological and research perspectives. There are several comprehensive reviews and textbooks about immunity to tuberculosis infection, particularly concentrating on genetic susceptibilities (2) and therapeutics (3). However, the unique contribution of this book is that it is the most contemporary resource, which draws on the expertise of 25 distinguished international specialists to provide a single authoritative book on the induction and maintenance of immunity against tuberculosis infection.

## The Challenge of Immunity to Tuberculosis

Infection by *Mycobacterium tuberculosis* is characterized by infiltration of alveolar macrophages, which is explored extensively within the book. By altering the intracellular environment of alveolar macrophages, the pathogen is able to replicate successfully within the inhospitable cellular environment. *Mycobacterium tuberculosis* is able to inhibit the induction of apoptotic pathways and autophagy within macrophages whilst initiating necrosis in order to evade host defenses and prevent the launch of an immune response against the pathogen. This presents a potential therapeutic target to induce immunity to tuberculosis infection. The book also discusses lipid mediators of pro-apoptotic and pro-necrotic pathways within macrophages, thus highlighting and delineating the importance of these pathways in ultimately determining whether successful immunity is stimulated against *Mycobacterium tuberculosis* infection or if clinical disease develops. Interestingly, alveolar macrophages are also target cells for HIV infection. The book discusses HIV and tuberculosis co-infection in several chapters, thus highlighting the importance of their relationship. The burden of HIV infection on host immune function substantially increases the risk of *Mycobacterium tuberculosis* infection and its progression to active disease; therefore, understanding the complex association between both infections is important for successfully tackling tuberculosis.

The induction of immunity to tuberculosis infection is mediated via CD4^+^ effector T cells and CD8^+^ T cells, which are discussed to a great extent within the book. Recently, there has been interest in the ability of γδ T cells to restrain tuberculosis infection as well (4). The book touches on this briefly; however, with fascinating developments occurring in this field, it would be interesting if it were expanded into a separate chapter. A particularly distinctive aspect to this book is that it discusses the relationship between regulatory T cells (Treg) and tuberculosis infection. Treg may play a role in limiting inflammation-mediated damage during infection; however, the book also notes that the induction of immunity to tuberculosis and infection clearance also expands CD4^+^ regulatory T cell (Treg) populations that express the transcription factor FOXP3. This is discussed in further detail in a chapter by Ryan P. Larson and Shahin Shafiani (5). Treg inhibit inflammatory processes and reduce the potency of effector responses to infection. Thus, Larson and Shafiani hypothesize that *Mycobacterium tuberculosis* is capable of hijacking Treg biology and promoting their expansion in order to serve the needs of the bacterium and avoid detection by the adaptive immune response. Very little is known about the mechanism by which *Mycobacterium tuberculosis* expands Treg; however, this could be a potential therapeutic target for improved management of tuberculosis. By inhibiting the factors that promote Treg expansion, potent effector responses can then be initiated by the adaptive immune response to clear infections. Interestingly, regulatory ability within the T cell population is not confined solely to CD4^+^ T cells. It has been shown that CD8^+^ T cells can also induce FOXP3 expression and become tolerogenic after antigenic stimulation (6). Therefore, it would be intriguing if further research will identify a relationship between CD8^+^ Treg and tuberculosis infection, and determine if these cells contribute to the pathogenesis of disease.

## Strategies to Investigate and Induce Immunity

Understanding host and bacterial pathways involved in tuberculosis infection is essential for identifying novel measures for combating infection. The book explores tuberculosis research involving zebra fish and granuloma formation by an infected host. Granulomas are organized aggregates of mature macrophages, which are critical for host immunity to tuberculosis infection. The book also examines the very important field of vaccination research and immunization strategies against tuberculosis. Effective vaccination is essential for restraining the spread of *Mycobacterium tuberculosis* infection and underlies effective immunity against the pathogen. This book is exceptional in that it combines knowledge on the mechanism of induction of humoral immunity to tuberculosis with recent research developments, which take into account the route of immunization to enhance vaccine-elicited immunity. A greater understanding of these processes, which improve the efficacy of vaccines and thus boost immunity to infection, will be very important in limiting the global spread of *Mycobacterium tuberculosis* infection.

In conclusion, Divangahi has edited an excellent and comprehensive book that brings together the expertise of eminent specialists within the field of immunity to tuberculosis. The book explores the epidemiology and biology of *Mycobacterium tuberculosis* infection, whilst investigating therapeutic applications and immunization strategies that may contribute significantly to reduce the global burden of disease.
